# Explicit versus implicit social cognition testing in autism spectrum disorder

**DOI:** 10.1177/1362361313492393

**Published:** 2014-08

**Authors:** Björn Callenmark, Lars Kjellin, Louise Rönnqvist, Sven Bölte

**Affiliations:** 1 Stockholm County Council, Sweden; 2 Örebro County Council, Sweden; 3 Örebro University, Sweden; 4 Umeå University, Sweden; 5 Karolinska Institutet, Sweden

**Keywords:** Asperger’s syndrome, assessment, mentalizing, neurodevelopmental disorders, psychometrics, theory of mind

## Abstract

Although autism spectrum disorder is defined by reciprocal social-communication impairments, several studies have found no evidence for altered social cognition test performance. This study examined explicit (i.e. prompted) and implicit (i.e. spontaneous) variants of social cognition testing in autism spectrum disorder. A sample of 19 adolescents with autism spectrum disorder and 19 carefully matched typically developing controls completed the Dewey Story Test. ‘Explicit’ (multiple-choice answering format) and ‘implicit’ (free interview) measures of social cognition were obtained. Autism spectrum disorder participants did not differ from controls regarding explicit social cognition performance. However, the autism spectrum disorder group performed more poorly than controls on implicit social cognition performance in terms of spontaneous perspective taking and social awareness. Findings suggest that social cognition alterations in autism spectrum disorder are primarily implicit in nature and that an apparent absence of social cognition difficulties on certain tests using rather explicit testing formats does not necessarily mean social cognition typicality in autism spectrum disorder.

## Introduction

Qualitative impairments in reciprocal social interaction and communication form two elements of the autism triad. Robust evidence indicates that atypical social cognition accounts for a substantial proportion of such behaviour difficulties in autism spectrum disorder (ASD) (Frith and Happé, 2005; Happé et al., 2006). The term ‘social cognition’ generally refers to the perception, processing and interpretation of information related to social interaction with regard to conspecifics (Brothers, 1990). In ASD, the process of acquisition of inherent social cognition is derailed early on, likely as a result of reduced salience of social stimuli and concomitant enactment of socially irrelevant aspects of the environment (Klin et al., 2003). Social cognition is composed of dual processes: explicit (controlled, slow and increasingly conscious; Frith and Frith, 2008; Satpute and Lieberman, 2006) and implicit cognition (spontaneous, fast and increasingly unconscious; Spunt and Lieberman, 2013), with the implicit system preceding the explicit system (Apperly and Butterfill, 2009; Low and Perner, 2012; Satpute and Lieberman, 2006). Language and executive functions, such as the ability to generalize (to encode and compare abstraction across contexts), are important to combine and harmonize implicit and explicit reasoning in social cognition (Low and Perner, 2012). It is presumed that individuals on the autism spectrum lack implicit social cognition but may acquire explicit skills through learning and experience (Frith, 2004). Normally, they do not automatically attend to socially relevant information but might be able to process the social information when their attention is navigated towards it (Senju, 2012a). In controlled assessment settings including explicit instructions, individuals with ASD often perform better than in spontaneously occurring natural situations (Paul and Cohen, 1985). Dewey (1991) postulated that social cognition in ASD is rather based on trying to follow static rules and logical reasoning than on social awareness and intuition. Frith and Happé (1999) described the social cognition strategies used by individuals with ASD as slow and consciously calculating.

Although social cognition alterations are a hallmark of ASD in a substantial minority of studies, individuals with ASD show a performance in social cognition largely comparable to typically developing (TD) or psychiatric controls, including affect processing tasks, indicating no atypicality or deficit (e.g. Bowler, 1992; Buitelaar et al., 1999; Castelli, 2005; Ozonoff et al., 1990). This seeming paradox might be related to how much social cognition is operationalized explicitly or implicitly, with an increasing explicitness being associated with an increasing likelihood of passing the tests. As Klin et al. (2004) and Ponnet et al. (2004) have pointed out, social cognition tests in the form of social situations often contain in narrative form all necessary facts for making a socially adequate decision. In addition, Dewrang (2011) argued that some of the stories used in social cognition tests are logical in the composition of the plot and that the actions in the stories happen one at a time. In controlled assessment conditions with explicit instructions and limited behavioural options, individuals with ASD may be able to perform better than in unstructured situations in which they must act rather freely and spontaneously (Paul and Cohen, 1985).

Differentiating between spontaneous (i.e. implicit) and elicited (i.e. explicit) social cognition thus might be important when testing individuals with ASD. Based on the evidence described above, we hypothesized that individuals with ASD, when explicitly prompted (by using a multiple-choice format), would reason around descriptions of social situations no differently from TD individuals. In contrast, we hypothesized that individuals with ASD, when judging social situations in a more free, spontaneous unstructured fashion, would give explanations differing from those of TD controls in terms of spontaneous perspective taking and implicit social awareness.

## Methods

### Participants

The sample comprised 19 adolescents with ASD (6 females, 13 males) with a mean age of 15.1 years (standard deviation (*SD*) = 1.6 years, range = 13–18 years) and a mean vocabulary scaled score on the Swedish versions of the *Wechsler Intelligence Scale for Children–Third Edition* (*WISC-III*; Wechsler, 1999), *WISC–Fourth Edition* (*WISC-IV*, Wechsler, 2007) or *Wechsler Adult Intelligence Scale–Third Edition* (*WAIS-III*; Wechsler, 2003) of 8.8 (*SD* = 2.6, range = 4–14). This group was carefully matched pairwise with 19 TD participants for sex (6 females, 13 males), age (mean = 15.3 years, *SD* = 1.7 years, range = 13–18 years) and vocabulary (mean = 8.8, *SD* = 2.6, range = 4–14). These highly comparable parallel cohorts were selected from a larger sample of 20 individuals with ASD and 73 with typical development. Informed consent was collected from all participants and their parents. ASD participants were recruited from three child and adolescent neuropsychiatric departments in Örebro County (central Sweden). ASD diagnoses were clinical *Diagnostic and Statistical Manual of Mental Disorders* (4th ed., text rev.; *DSM-IV-TR*) consensus diagnoses by child psychiatrists, child psychologists and paediatricians corroborated by results (cut-off met for ASD) from the Swedish version of the *Autism Diagnostic Observation Schedule* (*ADOS*) (Lord et al., 1999); exceptions were three participants who were diagnosed clinically only, without the availability of *ADOS* scores. Single *DSM-IV-TR* diagnoses in the ASD sample were autistic disorder in 8, Asperger’s disorder in 5 and pervasive developmental disorder–not otherwise specified in 7. Control participants were recruited from two secondary schools and one high school in the city of Örebro.

### Instrument and procedure

To examine explicit versus implicit social cognition in ASD, we used the Dewey Story Test (1991). It is a vignette-based test requiring that participants depict violations of social norms using eight sample situations. The test is widely used in Scandinavian countries and beyond in both clinical practice and research (e.g. Blair and Cipolotti, 2000; Dewrang, 2011; Ellis et al., 1994; Söderstrand and Almkvist, 2012; Vermeulen, 2002). To measure explicit social cognition, we used the standard scores of the Dewey Story Test. Here, the test-takers are explicitly asked to rate how they thought most people would judge the described behaviour in the stories if they witnessed it, according to a multiple-choice answering format. To measure implicit social cognition processing, we introduced the concepts of spontaneous perspective taking and implicit social awareness to the Dewey administration and scoring. Spontaneous perspective taking is the ability to explain behaviour using other people’s mental states such as thoughts, feelings and desires, without being explicitly prompted to (Senju, 2012a, 2012b). Implicit social awareness refers to the internalization of social rules and norms that create a cognitive shortcut when predicting a person’s behaviour in a certain situation (Baird and Baldwin, 2001; Low and Perner, 2012; Povinelli and Vonk, 2004).

A Swedish translation of the Dewey Story Test was used (Dewey, 1998). Slight changes were introduced compared to the English original to better fit Swedish culture and language: for example, the names of people appearing in the situations were modified to names commonly given in Sweden; in vignette 4, the currency was changed to Swedish Crowns; and in vignette 3, the probable cause of a screaming child was changed from an open safety pin in the diaper to a wet diaper. The eight Dewey Story Test stories are divided into 24 subsections (1.1, 1.2, etc. to 8.6). For each section, the participant has to decide using a multiple-choice format how most people would perceive the behaviour of a protagonist, using the following categories: (a) fairly normal, (b) rather strange, (c) very eccentric and (d) shocking. The Dewey Story Test and the test instructions are presented in Appendix 1. The test was administered individually in a quiet room, with the stories read aloud to the participants who could follow the story in their own copy of the text.

Explicit social cognition was measured using the Dewey Story Test multiple-choice (4 choices) total score for the 24 subsections, following the scoring principles first applied by Ellis et al. (1994) and later used by Blair and Cipolotti (2000), Vermeulen (2002), Dewrang (2011) and Söderstrand and Almkvist (2012). Here, deviance scores are generated, with increasing scores indicating decreasing performance. Categories are first ranked depending on answer patterns. The responses preferred by a majority of the TD participants are viewed as correct, equalling a score of 0. The cut-off criterion for qualifying as a substantially supported answer was set to a response ratio of 30% (Söderstrand and Almkvist, 2012). The category with second highest support received a deviation score of 1, and the other two categories received deviation scores of 2 and 3 points, respectively. For the Dewey’s total deviance score, the maximum is 57. The test–retest reliability (Pearson *r*) for this total score within the Swedish version of the Dewey Story Test was examined using responses from 10 participants in the TD group, reaching *r*_tt_ = .53 after an average interval of 5.4 months.

To assess implicit social cognition in terms of spontaneous perspective taking and social awareness on the Dewey Story Test, a method derived from Vermeulen (2002) was used. After the explicit assessment using the multiple-choice format, the participants were freely interviewed for 5 min about why they thought most people would perceive the protagonist’s behaviour in a certain way (e.g. ‘Why do you think this behaviour is fairly normal/rather strange/very eccentric/shocking?’). These responses were tape-recorded and transcribed verbatim. Originally, Vermeulen (2002) categorized the answers in six different ways: no motivation, references to general rule, references to self or own experience, references to physical reality in the story, references to the main character’s perspective and references to a minor character’s perspective. From this scheme, we extracted two new categories to simplify the scoring. First, ‘references to general rule’ and ‘references to physical reality in the story’ were collapsed to create a new category labelled ‘implicit social awareness’, containing references about social norms and rules, but no perspective taking. It was defined by comments such as ‘it is not normal to’ or ‘you cannot do that’. Second, ‘references to the main character’s perspective’ and ‘references to a minor character’s perspective’ were collapsed to create a new category labelled ‘spontaneous perspective taking’, containing different forms of perspective taking and how people were feeling and thinking, but no references to social norms and rules. It was defined by comments such as ‘he wants to know her name’ or ‘it is embarrassing for the other person’. A more complete list of common answers operationalizing the categories for each of the 24 subsections of the Dewey’s test is provided in Appendix 2.

The total score for each category was defined by the number of times the participant used the category. When overlapping references that fit both categories were given, a score was assigned for each of the categories separately. For spontaneous perspective taking, the maximum score was 48 (if a participant used both a main and a minor character perspective taking in all sections), and for implicit social awareness, the maximum score was 24 (if a participant gave at least one indication of using an implicit social norm in all sections).

The inter-rater reliability for rating spontaneous perspective taking and implicit social awareness was examined using intra-class correlation between ratings. This correlation was made by an independent layperson blinded to the data, who rated five interview transcripts each from the ASD group and the TD group and compared those ratings with the examiner’s ratings on these cases (720 vs 720 ratings in total). The agreement between the two raters was substantial (*r*_ic_ = .70, confidence interval = 0.66–0.74). The test–retest reliability (Pearson *r*) for spontaneous perspective taking and implicit social awareness scores for 10 TD participants, after an average interval of 5.4 months, reached *r*_tt_ = .68 (*p* = .01) and *r*_tt_ = .59 (*p* = .04), respectively.

### Data analyses

To examine explicit versus implicit social cognition performance on the Dewey Story Test between the one-by-one matched ASD and TD groups, *t*-tests for paired samples were run. Primary assumptions of parametric testing were fulfilled (Gaussian distribution, homogeneity of (error) variances). An alpha of 5% was adopted, but because of the rather small sample sizes and the associated risk for type 2 errors with regard to small effects, trends (*p* < .10) were also interpreted. Between-group differences were not expected for explicit social cognition (multiple-choice total score) but were predicted for implicit social cognition (spontaneous perspective taking and implicit social awareness scores based on free interview). Given the sample size of this study and the *alpha* (.05), the test power (1-*beta*) for detecting significant group mean differences between the samples using a dependent *t*-statistic was .33 for a small (*d* = .20), .91 for a medium (*d* = .50) and .99 for a large (*d* = 80) effect. Correlations, partialled out for verbal abilities (vocabulary) between the explicit and implicit scores and ADOS scores in the ASD sample, were calculated if informative for understanding the Dewey Story Test findings.

## Results

**Figure 1.**
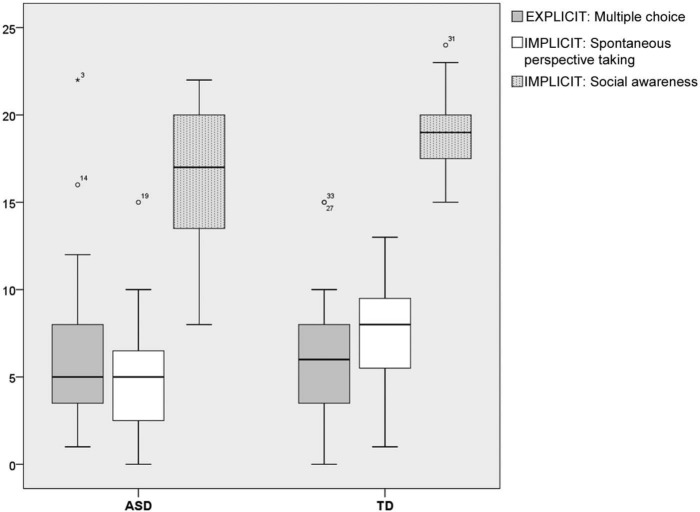
Box plot for Dewey’s Test performance in explicit social cognition (Dewey’s total multiple-choice score) and implicit social cognition (spontaneous perspective taking and social awareness) between the autism spectrum disorder (ASD) and typically developing (TD) control groups.

In line with our hypotheses, explicit social cognition (Dewey’s multiple-choice total score) did not differ between the ASD (mean = 6.9, *SD* = 5.2, range = 1–22) and TD groups (mean = 6.2, *SD* = 4.1, range = 0–15) (*t* = .694, *p* = .50) (Figure 1). In addition, as expected, the ASD group differed from the control group in the implicit social cognition measures. Spontaneous perspective-taking scores were significantly lower and on average 5.1 (*SD* = 3.6, range = 0–15) in ASD and 7.4 in controls (*SD* = 3.3, range = 1–13) (*t* = −2.2, *p* = .04). For implicit social awareness, there was a trend (*t* = −1.92; *p* = .07) for decreased performance in the ASD group (mean = 16.4, *SD* = 4.4, range = 8–22) compared to the control group (mean = 18.8, *SD* = 2.5, range = 15–24). In the ASD sample (*n* = 16, three *ADOS* missing), *ADOS* scores for social interaction and communication correlated positively with the Dewey’s multiple-choice total score explicit social cognition measure (*r* =.53 and .35, *p* < .03), but negatively with spontaneous perspective taking (*r* =.−32 and −.40, *p* < .03) and implicit social cognition measure.

## Discussion

Consistent with the hypotheses of this study, ASD and TD individuals did not differ in explicit social cognition as operationalized by the multiple-choice prompting on the Dewey’s Story Test. This finding is consistent with the results of Ellis et al. (1994), Söderstrand (2004) and Dewrang (2011), who also found no differences between controls and ASD participants on the Dewey’s Story Test for the multiple-choice total score. Moreover, in line with our expectations, differences emerged with regard to implicit social cognition tasks, operationalized as free verbal judgement of the Dewey’s social situations in terms of spontaneous perspective taking and implicit social awareness. Although individuals with ASD gave indications of spontaneous perspective taking and implicit social awareness, both were present more consistently in the TD group. Because samples were carefully matched, these differences are likely not attributable to differences in language abilities, age or sex.

Similar to our findings, Nah and Poon (2010) also observed differences between explicit and implicit social cognition using closed and open item formats when testing for social cognition skills. They examined children ages 9 to 13 years with ASD and a group of age/sex-matched controls using Dewey Story Test–like vignettes. The children with ASD overall rated the vignettes no differently from their TD peers when using closed categorical item response formats. However, in the interviews of the children regarding how they reached their decisions, the ASD group generated many inappropriate responses, including failure to respond at all.

Interestingly, in the current ASD group, *ADOS* scores for social interaction and communication problems correlated positively with the explicit social cognition measure on the Dewey Story Test with a closed answering format while implicit social processing in terms of spontaneous perspective taking showed a negative association with *ADOS* scores. A possible inference is that the explicit social cognition measures not only show limited sensitivity to social cognition atypicalities in ASD but also themselves measure some form of autistic behaviour. The latter is consistent with a multitude of findings indicating that explicit instructions and demands are beneficial for the performance of individuals with ASD, compared to more naturalistic situations requiring active flexible responses (Paul and Cohen, 1985; Senju, 2012a).

Our findings support the notion that explicit, prompted answer formats may decrease the complexity of social cognition demands and facilitate the use of compensatory strategies (Dewrang, 2011; Klin et al., 2004; Ponnet et al., 2004). Such formats surely have strengths with regard to objectivity, economy and standardization but probably at the price of decreased sensitivity to social cognition alterations in ASD. Open formats, such as those used in this study, probably create a more naturalistic way of evaluating social cognition in verbal individuals with ASD. Inter-rater and retest reliability analyses showed good interpersonal fidelity and stability for scoring these unstructured assessments.

This study has several limitations, including a rather small sample size and a lack of inclusion of a more established social cognition measure to explore convergent validity of the Dewey Story Test measures (e.g. Reading Mind in the Eyes (Baron-Cohen et al., 2001) or Strange Stories (Happé, 1994)). Perhaps most notable is the immature stage of our classification of the Dewey’s Story Test multiple-choice performance embodying explicit social cognition skills, and the interview-based perspective taking and social awareness measures of implicit social cognition. Although labelling the multiple-choice measure as explicit is quite straightforward because of its demand for an active and conscious process of selecting answers from a given choice, the labelling of the free interview-based measures as implicit is surely arguable. A general definition of implicit social cognition is unconscious, automatic, fast and not requiring attention or verbal report at the level of psychological test performance. Usually, to assess implicit social cognition, participants perform an apparently irrelevant task (e.g. judging the gender or attractiveness of a person) during collection of an indirect measure such as eye movement, reaction times or neural responses. This design does not apply for the implicit measure used in this study. Thus, ‘implicit’ here is defined rather by an in-depth capacity to demonstrate theory of mind skills, convey comprehension of social life and reason about and spontaneously elaborate on a given multiple-choice reply.

In conclusion, when explicit social cognition was assessed using the Dewey Story Test and a multiple-choice answering format, ASD and TD individuals did not differ in how they thought most people would judge social behaviour. However, ASD and controls did differ when tested with a more complex implicit social cognition measure assessing spontaneous perspective taking and implicit social awareness. The use of categorical, prompted answering formats is likely to decrease the complexity of social cognition demands and might facilitate the use of compensatory strategies, leading to unremarkable social cognition performance in ASD. Rating a free verbal report might create a more naturalistic way of evaluating implicit social understanding.

## Appendix 1

### Dewey Story Test

#### Test instructions

In the following stories, some parts are in *italics*. Rate the behaviour that is illustrated by the portion in italics according to how you think most people would judge that behaviour if they witnessed it. Use the following scale:

A: Fairly normal behaviour in that situation
B: Rather strange behaviour in that situation
C: Very eccentric behaviour in that situation
D: Shocking behaviour in that situation

After each section of the story, you will be asked to explain why you made that particular rating.

### Story 1: In the supermarket

1:1 The market where Robert always shopped had a small sign on the door that read BARE FEET PROHIBITED IN THIS STORE BY STATE LAW. One summer day, Robert saw a pretty girl enter the store without shoes. She seemed to be about his age, 20, with long hair and an old-fashioned dress reaching to her ankles. Robert wanted to warn her about the sign, but he was afraid to speak to her. Unpleasant things happened if he tried to talk to strange girls. Finally, he decided he might be able to shield her feet from being seen by the manager. *He pushed his cart close behind hers down aisle after aisle.*
1:2 Once or twice, the girl looked back at him with a cross expression. Suddenly, she wheeled into the quick-check lane with 12 items in her basket although the sign said ‘FOR TEN OR FEWER ITEMS’.
1:3 Robert was more upset than ever. He thought this pretty girl was tempting fate by breaking another rule. When the check-out clerk let her through without comment, Robert finally relaxed. *Just then, the barefoot girl turned and said to him, ‘I don’t know why you are following me, but buzz off or I’ll call the police!’*

### Story 2: In the elevator

2:1 Kalle, 23, had been out of work for several months. On this day, his hopes were high because he was on his way to apply for a job that seemed just right for him. As Kalle rode the elevator to his interview, *a stranger said pleasantly, ‘Nice day, isn’t it?’*
2:2 Just then, Kalle happened to see his reflection in a mirror by the elevator buttons. His hair was sticking up in a peculiar way, and he had no comb with him. *He turned to the friendly stranger and asked, ‘Do you have a comb I could borrow for a minute, please?’*

### Story 3: In the park

3:1 Tommy, age 25, was a file clerk who worked in an office in the city. At noon, he took his lunch to a small park and sat on a sunny bench to eat. *Often, he tore part of his sandwich into bits, scattering it on the ground for the pigeons.*
3:2 One day, when he came to his favourite bench, a baby carriage was parked beside it. Tommy noticed a young woman was swinging an older child nearby. The baby in the carriage began to cry, but the mother did not hear this because the swing was squeaking. Now Tommy had learned that when his baby nephew screamed, sometimes this meant his diaper was wet. (original: that a pin in his diaper had opened.) *Rather than bother the mother in the park, Tommy quickly checked the baby’s clothing to see whether the diaper was wet. (original: he could feel an open pin.)*

### Story 4: The forgotten name

4:1 Paul, 23, had a little shop where he renovated old furniture. Sometimes, a customer would ask to have some work done in her home. On one such occasion, an elderly lady called him to stain a scratch on her desk. *Unfortunately, Paul forgot to jot down her name when he wrote down the address.*
4:2 The lady greeted him warmly at her door, saying ‘Come right in, Paul, I have heard that your work is good’. Ashamed because he had forgotten her name, *Paul waited until she left the room and peeked into a drawer.*
4:3 Sure enough, he found some letters addressed to Mrs Isabel DeWitt, and this jogged his memory. Satisfied, Paul shut the drawer without disturbing anything and soon had the scratch nicely refinished. When the lady of the house saw it, she said, ‘That’s perfect! How much do I owe you, Paul?’ *He replied, ‘It did not take very long, so 100 kr will be fine, Isabel’.*

### Story 5: In the airplane

5:1 Emilia, age 19, overslept on the morning of her airplane trip. When she woke up, there was just enough time to dress and get to the airport, so *she skipped her breakfast.*
5:2 At noon, the flight attendant came around with lunch, but Emilia was so hungry by then that one portion did not satisfy her. She watched a little girl across the aisle toy with her food, complaining, ‘I can’t eat it’. Apparently, the father didn’t want any more, because he told the child to just leave it. *Emilia leaned across the aisle and said, ‘If your little girl doesn’t want her tray, can you pass it over for me?’*

### Story 6: The dinner invitation

6:1 Roger, 22, lived in a rented room alone. He was quite a nervous person, but it seemed to him that he felt better if he ate every two hours and limited his diet to certain foods. One day, a lady called and invited him to dinner, explaining that she was a friend of his parents. Roger gladly accepted. *However, he warned his hostess that he ate no meat and would like his vegetables served unsalted.*
6:2 When Roger arrived at the appointed time, he recalled that he had not eaten for two hours. *Without wasting any time, even before the introductions, he asked his hostess when dinner would be served*.
6:3 She replied that it would be about an hour before the meal would be ready. *Hearing this, Roger opened his briefcase, removed an apple and some nuts, and promptly ate them*.
6:4 After that, he was introduced to the family, and they sat talking for an hour. Just before dinner, the hostess showed him an attractive platter of fruits and vegetables, asking whether it looked like enough. ‘*It looks fine, thank you’, Roger said, ‘but if you don’t mind, I will wait another hour to eat. I just had some food an hour ago’.*

### Story 7: Forbidden foods

7:1 Elizabeth had been diabetic most of her life. Doctors told her that careful attention to diet was necessary to avoid serious complications. When she was invited to someone’s home for a meal, she explained her problem in advance. *But at large gatherings, she handled the matter herself by avoiding forbidden foods or leaving them untouched on her plate.*
7:2 On such occasions, she did not mention her medical condition unless somebody urged forbidden food on her, in which case she said, ‘No thanks, I’m diabetic’. At some parties, there was not much she could eat, and *in such situations, she enjoyed the conversation and companionship, waiting until she returned home to eat the food she was allowed to eat.*

### Story 8: The lunch-time nap

8:1 Frank found employment at the age of 19 with a company that cared for people’s yards. He carried his lunch with him in a box. *At noon, Frank washed his hands under the hose and sat in a shady part of the yard to eat.*
8:2 Because he was allowed an hour for lunch, *he sometimes snatched a quick nap by curling up behind a bush*.
8:3 One day, it began to rain at noon. Frank knocked on the door and asked permission to eat inside. The lady said he could come in, and because she was busy with her children, he decided not to bother her further. *He located the bathroom by himself and washed his hands.*
8:4 *Then he found the dining room by himself and ate his lunch*.
8:5 *He cleaned the crumbs from the table and looked around the house for a place to rest*.
8:6 The living room carpet was thick, *so he decided to curl up for his nap behind a large chair*.

## Appendix 2

### Common answers

**Table:**

	Implicit social awareness	Spontaneous perspective taking
1.1	It is not normal, you can’t do that	He liked her/thinks she is nice and tried to protect her/help her
	Strange to follow people everywhere	He was probably afraid to talk to her
	You must have shoes on in the shop	She thought it was odd/uncomfortable/got very scared of it
1.2	Strange/common not to follow the rules	She was afraid/she likes to break rules
	Because she had not looked at the sign	She went there to escape him/she would like to come out sooner because she feels stressed
		She was not aware of how many items she had
1.3	Normal reaction, you can’t follow people around like that	He likes her, he wanted to help
	It is dangerous to yell at someone whom you think is weird	She felt threatened/scared/angry/uncomfortable/unsure
	The police can help/you don’t call the police	She does not know who he is, she wonders why/may feel that he pursued her
2.1	It is customary to do so/it is common/it’s polite to say ‘nice weather’	He seemed optimistic about the interview
	You do not see the weather in an elevator	He wanted to talk/be nice/he was nervous/thought it was beautiful weather
		The stranger/ was bored/wants to know him/wanted to be nice and have a chat in the elevator
2.2	It is normal/abnormal/common to do so	If he notices that the other person was nice, maybe he was not shy to ask for it
	The comb may have lice/few carry a comb	It is shocking to the person he asks to borrow from
	You have to have nice hair for an interview	Those at the interview might think he looks weird
3.1	It is normal/abnormal/It is customary to do so/Many do so	He liked the doves/It is annoying to those sitting next to him
3.2	It is not normal/You do not touch children whom you do not know	The mother could become angry/scared/misunderstand the situation
	It is normal, he has learned it as a routine	The mother might think that you were trying to steal the baby
		If I were a mother, I would go crazy
4.1	It is normal/abnormal/It’s easy to forget	So maybe he forgot the name, thinking of something else
		Maybe he was so stressed/became excited and had a lot to think about
		He knows the address/will remember it anyway
4.2	You should not dig into someone else’s private life	He was curious/felt a little awkward not knowing the name
		She might think he tried to steal/would get angry/He wants to know who she is
4.3	It’s normal/not normal	He wanted to show that he knew her name
	She addressed him with the name, so he did the same thing to her	She does not know that he was looking through a desk drawer
	You usually get paid when you have done a good job	She did not know that he did not know
5.1	It is common/not common	She was stressed/wanted to get more time/wanted to catch the plane
	It is more important to catch the plane	Maybe she thought she could eat something on the plane
	You can have breakfast on the plane	
5.2	You do not ask other people about their food	She was hungry/had heard their conversation
	You can get sick from eating others’ food	They were a bit shocked/scared/angry
	It is strange to take food from strangers	All the other people find it strange
6.1	It is normal/not normal	He wanted to feel good, he does not know who she is
	Because he is a vegetarian	Otherwise may get sad/ashamed
	It’s like being allergic, then you should also tell people	
6.2	It will be strange/One usually does not act like that	He was probably hungry/It’s not so fun to feel bad
		He thinks only of the food and not about her or the rest of the family
		She has invited him to her home and will be nice
6.3	If you’re hungry you must eat/You shouldn’t eat anything before dinner	He was hungry/nervous
	Fruit is not wrong to just eat, but the nuts?	They think that he doesn’t want to eat their food/that it was not good enough
		They understood that he was hungry/did not know his habits
6.4	One usually does not do so	He was unhappy/not hungry
	When you get invited to dinner, you eat with the family, you don’t sit by yourself and eat apples and nuts	She does not understand why he did not want anything
7.1	Because you usually tell if you have such a problem	She is ashamed
	It’s not healthy to eat stuff you shouldn’t, she can die from it	The hostess may get unhappy/may feel as if her disease is a burden
	It’s better to be safe than sorry	
7.2	Smart to wait until she comes home, it’s good	Then she’s very hungry/doesn’t bother them
	As a diabetic, you can die if you eat something else,	
	she falls ill	
	Would be antisocial to walk away	
8.1	That is normal since he works there/You have to eat lunch	Maybe he was hungry/wanted to sit in the sun
	It’s cleaner to wash your hands/not clean to wash your hands under the water hose	The owner will not get angry/might find it strange
		The owner should know that he is there if he hired him
8.2	It is common/not common	He is tired/enjoys it
	You don’t normally sleep in a bush/on the job	The owner may feel that he is not serious in his work
	If you’re tired you can sleep/If you do not disturb it’s ok	If I were the owner and I got there, I’d probably be pretty scared/surprised
8.3	Better to go in than sit out in the rain	He wanted to have clean hands/didn’t want to bother
	Strange to wash your hands without asking	She may think that he is snooping around
8.4	He had a lunch break and ate his lunch	He was hungry/He didn’t want to disturb her
		Maybe they thought he would eat it in the dining room
	He should have asked	Maybe he saw that she did not want to be interrupted
8.5	You should not rest in someone else’s house whom you do not know	He is tired/wants the rest afterwards
	If you are tired you should sleep/You should not sleep on the job	They will be surprised/not feel comfortable/if they find him sleeping
	Spontaneous perspective taking	She had not expected that he would sleep there too
8.6	You don’t do that/It’s uncommon	The lady will think that he fainted/died/was mentally disturbed
	He did not know the family	If I were the lady, I would be shocked
	You rest in the chair, not on the floor	
